# Rifaximin Use Is Associated With Prognosis and Recurrence Patterns in Spontaneous Bacterial Peritonitis: Exploratory Insights Into the Oral–Gut–Hepatic Axis

**DOI:** 10.1002/jgh3.70391

**Published:** 2026-04-02

**Authors:** Yoshihito Uchida, Naoto Soma, Shunsuke Yamada, Shohei Tsuji, Satsuki Ando, Kayoko Sugawara, Masamitsu Nakao, Nobuaki Nakayama, Yukinori Imai, Suguru Mizuno, Satoshi Mochida

**Affiliations:** ^1^ Department of Gastroenterology & Hepatology, Faculty of Medicine Saitama Medical University Saitama Japan

**Keywords:** liver cirrhosis, oral hygiene, refractory ascites, rifaximin (RFX), spontaneous bacterial peritonitis (SBP)

## Abstract

**Background:**

Spontaneous bacterial peritonitis (SBP) remains a life‐threatening complication in cirrhotic patients with refractory ascites. Evidence for rifaximin (RFX) in SBP prevention and prognosis is lacking in Japan. We investigated the effect of RFX on prognosis and recurrence of SBP and evaluated the prognostic role of oral health within the oral–gut–hepatic axis.

**Methods:**

This single‐center, retrospective study included 142 patients with a first SBP episode. Patients were stratified into an RFX‐treated group and a non‐RFX group. Outcomes included 90‐day survival, SBP recurrence, and cirrhosis‐related complications. Oral health status and acid‐suppressive therapy were also evaluated.

**Results:**

Forty‐seven patients received RFX. Ascitic culture positivity was comparable between groups, but RFX‐treated patients had fewer enteric bacteria and more oral flora (*p* = 0.0033). Ninety‐day survival was significantly higher with RFX (85.1% vs. 60.0%, *p* = 0.0023). The 12‐month recurrence rate was markedly lower in the RFX‐treated group (10.4% vs. 63.2%; HR 0.19, 95% CI 0.09–0.40). Oral contamination independently predicted overt hepatic encephalopathy (HR 2.56) and variceal rupture (HR 6.72). Denture use was an independent predictor of mortality (HR 2.04, 95% CI 1.23–3.38).

**Conclusion:**

Rifaximin use was associated with higher short‐term survival and lower SBP recurrence in cirrhotic patients with refractory ascites. Oral contamination and denture use were associated with adverse outcomes, providing novel clinical evidence for the oral–gut–hepatic axis. Integration of RFX therapy with oral hygiene interventions may offer a comprehensive strategy to improve prognosis in cirrhosis.

**Abbreviations**
ACLF: acute‐on‐chronic liver failure
AIH: autoimmune hepatitis
AKI: acute kidney injury
ALD: alcohol‐associated or ‐related liver disease
F2: form 2 or greater (esophagogastric varices grading)
H2RA: histamine H2‐receptor antagonist
HBV: hepatitis B virus
HCC: hepatocellular carcinoma
HCV: hepatitis C virus
HR: hazard ratio
HRS: hepatorenal syndrome
IQR: interquartile range
JSGE: Japanese Society of Gastroenterology
JSH: Japan Society of Hepatology
MASH: metabolic dysfunction‐associated steatohepatitis
MELD: model for end‐stage liver disease
OHAT: Oral Health Assessment Tool
OR: odds ratio
P‐CAB: potassium‐competitive acid blocker
PPI: proton pump inhibitor
RFX: rifaximin
SAAG: serum–ascites albumin gradient
SBP: spontaneous bacterial peritonitis

## 1 Introduction

Liver failure in patients with cirrhosis progresses along two distinct clinical courses: chronic decompensation and acute‐on‐chronic liver failure (ACLF) [1, 2]. Patients with decompensated cirrhosis or ACLF frequently develop complications associated with liver failure and portal hypertension, such as jaundice, ascites, hepatic encephalopathy, variceal bleeding, and acute kidney injury (AKI). Among these complications, spontaneous bacterial peritonitis (SBP) is a major concern. SBP is defined as a bacterial infection of the ascitic fluid in the absence of an evident intraabdominal source of infection, such as intestinal infection or abscess formation. It is difficult to diagnose in the early stages and may be overlooked in clinical practice despite its severe consequences [3, 4]. The 1‐year survival rate after the onset of SBP has been reported to be approximately 40%, with a high recurrence rate and poor prognosis [5]. The “Evidence‐based Clinical Practice Guidelines for Liver Cirrhosis 2020” issued by the Japanese Society of Gastroenterology (JSGE) and the Japan Society of Hepatology (JSH) recommend prophylactic antibiotics administration in cirrhotic patients with refractory ascites, depending on the risk of infection [3, 4]. However, in Japan, prophylactic antimicrobial therapy is not covered by the national health insurance system, and its routine use is therefore limited.

Rifaximin (RFX) is a nonabsorbed, broad‐spectrum oral antibiotic that is active against both gram‐positive and gram‐negative aerobic and anaerobic organisms. It suppresses intraluminal ammonia‐producing bacteria, thereby reducing systemic ammonia levels. Consequently, RFX is widely used in the management of hepatic encephalopathy [6, 7]. Furthermore, several reports have suggested that RFX may also be effective in preventing the occurrence and recurrence of SBP in patients with refractory ascites [8]. However, its clinical efficacy in Japanese patients has not been elucidated.

On the other hand, long‐term administration of proton pump inhibitors (PPIs) in cirrhotic patients with ascites reduces the gastric acid barrier, promotes bacterial overgrowth, and alters the gut microbiota. These changes may facilitate bacterial translocation from the intestine to the peritoneal cavity, leading to the development of SBP [9]. Recently, alterations in the salivary microbiome and oral inflammation have been reported in patients with cirrhosis, introducing the concept of an “oral–gut–hepatic axis” [10, 11]. Indeed, Bajaj et al. have reported that appropriate periodontal care can reduce the risk of hepatic encephalopathy and infectious complications in patients with cirrhosis [12].

Therefore, to establish effective preventive and therapeutic strategies for SBP, the present study evaluated the effects of RFX administration, acid secretion inhibitors, and the oral environment on the pathophysiology and prognosis of SBP in patients with refractory ascites associated with cirrhosis.

## 2 Patients and Methods

### 2.1 Patients and Study Design

This single‐center retrospective observational study was conducted at Saitama Medical University Hospital (Moroyama, Saitama, Japan). Consecutive patients with cirrhosis and refractory ascites who experienced the first episode of SBP between January 2017 and October 2024 were eligible. Patients were divided into an RFX‐treated group and a non‐RFX group, and clinical characteristics at SBP onset as well as 90‐day prognosis were analyzed. Additionally, among patients who followed for more than 90 days after the first SBP episode, factors associated with SBP recurrence and long‐term prognosis were analyzed. Notably, observation was terminated at the point of RFX initiation when evaluating SBP recurrence. RFX was prescribed primarily for the treatment and prevention of hepatic encephalopathy, not for SBP itself. Patients were classified according to RFX use at the time of the first SBP episode (RFX group: patients already receiving RFX at SBP onset vs. non‐RFX group). Acid‐suppressive therapy was categorized as histamine H2‐receptor antagonist (H2RA), proton‐pump inhibitor (PPI), and potassium‐competitive acid blocker (P‐CAB).

### 2.2 Diagnosis of SBP

The diagnosis of SBP was based on the “Evidence‐based Clinical Practice Guidelines for Liver Cirrhosis 2020” issued by the JSGE and the JSH; SBP was defined as either in ascitic fluid culture or a neutrophil count in ascitic fluid ≥ 250/mm^3^ [3, 4]. Bacteria detected in ascitic fluid cultures were categorized into enteric bacteria, oral bacteria, and skin commensals according to their primary ecological niche based on established microbiological literature. A complete list of species and the rationale for classification is provided in Table S1.

### 2.3 Oral Health Status

Oral conditions at admission were evaluated retrospectively using medical records, focusing on denture use and oral contamination. Denture use was self‐reported by patients. Oral contamination was assessed using a chart‐based reconstruction of the Oral Health Assessment Tool (OHAT) framework [13].

In routine clinical practice at our institution, oral status is assessed by nursing staff at hospital admission as part of standard nursing evaluation. In this assessment, pathological findings in items such as “lips,” “tongue,” “gingivae/mucous membranes,” “saliva,” “oral cleanliness,” or “dental pain,” and/or noted abnormalities or changes in “natural teeth” or “dentures” prompt documentation indicating that oral care is required.

Based on this practice, OHAT‐related information was retrospectively reconstructed by the investigators from medical and nursing records documented at admission. “Oral contamination” was operationally defined as cases in which such documentation indicating the need for oral care was present, reflecting clinically relevant oral hygiene impairment.

### 2.4 Definitions of Renal Complications

Hepatorenal syndrome–acute kidney injury (HRS–AKI) was diagnosed according to the International Club of Ascites (ICA) criteria as documented in the medical records. AKI was defined by changes in serum creatinine after exclusion of other causes of renal dysfunction and lack of response to standard initial management.

### 2.5 Ethical Considerations

This study conformed to the ethical guidelines of the Declaration of Helsinki and was conducted with the approval of the Institutional Review Board of Saitama Medical University Hospital (2024‐094). Informed consent was obtained through an opt‐out process on the website of Saitama Medical University Hospital (https://saitama‐med.bvits.com/rinri/publish_document.aspx?ID=3596).

### 2.6 Statistical Analysis

Categorical variables were compared using either Fisher's exact test or the chi‐squared test, as appropriate. Continuous variables were analyzed using the Mann–Whitney U‐test. To identify predictors of 90‐day mortality following SBP, multiple logistic regression analysis was conducted. In patients with follow‐up beyond 90 days after SBP onset, the cumulative incidence of SBP recurrence and cirrhosis‐related events was analyzed using Gray's test, treating death as a competing risk. Fine–Gray sub‐distribution hazard models were used to evaluate factors associated with these outcomes. For the SBP recurrence analysis, the patients were classified into two groups based on the use of RFX at the time of the first SBP episode (RFX vs. non‐RFX). Since 8 of the 57 patients in the non‐RFX group initiated RFX during the follow‐up period because of the development of hepatic encephalopathy, the follow‐up period was censored at the time of RFX initiation to avoid misclassification of events occurring after the initiation of RFX as being under non‐RFX exposure. Death was regarded as a competing risk. Long‐term survival was assessed using the Kaplan–Meier method, and prognostic factors were analyzed using a Cox proportional hazards model. Survival curves were compared using the log‐rank test. All statistical tests were two‐tailed, and a *p* value of < 0.05 was considered statistically significant. For secondary group‐wise and subgroup comparisons (e.g., comparisons among H2RA, PPI, and P‐CAB groups), no formal adjustment for multiple testing was performed; these results were considered exploratory and should be interpreted cautiously. Statistical analyses were performed using EZR version 1.68 (Saitama Medical Center, Jichi Medical University, Saitama, Japan), which is a graphical user interface for R [14].

## 3 Result

### 3.1 Demographic Characteristics and Clinical Features of 142 Patients With Liver Cirrhosis at the Onset of SBP

A total of 142 patients with liver cirrhosis who developed the first episode of SBP were included (Table 1). The cohort consisted of 96 men (67.6%) and 46 women (32.4%), with a median age of 69 years (interquartile range [IQR], 57–76 years). The etiologies of cirrhosis were hepatitis C virus (HCV) in 37 patients (26.1%) and hepatitis B virus (HBV) in 3 patients (2.1%). Other caused included alcohol‐associated or ‐related liver disease (ALD) in 56 patients (39.4%), metabolic dysfunction‐associated steatohepatitis (MASH) in 23 (16.2%), autoimmune hepatitis (AIH) or primary biliary cholangitis (PBC) in 15 (10.6%), and others in 8 patients (5.6%), respectively.

**TABLE 1:** Baseline demographic and clinical characteristics of 142 cirrhotic patients at the onset of spontaneous bacterial peritonitis.

Variables	Total (*n* = 142)	The RFX‐treated group (*n* = 95; 66.9%)	The non‐RFX group (*n* = 47; 33.1%)	*p*
Age, years	69 (57–76)	70 (57–76)	64 (56–75)	0.4729
Sex, *n* (%)				0.3419
Men	96 (67.6)	67 (70.5)	29 (61.7)	
Women	46 (32.4)	28 (29.5)	18 (38.3)	
Etiology, *n* (%)				0.1033
HCV	37 (26.1)	24 (25.3)	13 (27.7)	
HBV	3 (2.1)	3 (3.2)	0 (0.0)	
ALD	56 (39.4)	42 (44.2)	14 (29.8)	
MASH	23 (16.2)	10 (10.5)	13 (27.7)	
AIH/PBC	15 (10.6)	10 (10.5)	5 (10.6)	
Others	8 (5.6)	06 (6.3)	2 (4.3)	
Child‐Pugh score	10 (9–12)	10 (9–12)	11 (10–13)	0.0140
Child‐Pugh class, *n* (%)				0.0087
B	38 (26.8)	32 (33.7)	6 (12.8)	
C	104 (73.2)	63 (66.3)	41 (87.2)	
SAAG, g/dL	1.9 (1.6–2.3)	1.9 (1.6–2.2)	2.0 (1.7–2.5)	0.0755
HCC present, *n* (%)	41 (28.9)	31 (32.6)	10 (21.3)	0.1747
Esophagogastric varices ≥ F2, *n* (%)	84 (59.2)	48 (50.5)	36 (76.7)	0.0036
Denture use, *n* (%)	52 (36.9)	40 (42.6)	12 (25.5)	0.0639
Oral contamination present, *n* (%)	5 (3.5)	3 (3.2)	2 (4.3)	1.0000
Acid‐suppressive therapy, *n* (%)				0.4802
H2RA	10 (7.1)	6 (6.3)	4 (8.5)	
PPI	100 (70.4)	70 (73.7)	30 (63.8)	
P‐CAB	32 (22.5)	19 (20.0)	13 (27.7)	

Abbreviations: AIH: autoimmune hepatitis, ALD: alcohol‐associated or ‐related liver disease, F2: form 2 or greater (esophagogastric varices grading), H2RA: histamine H2‐receptor antagonist, HBV: hepatitis B virus, HCC: hepatocellular carcinoma, HCV: hepatitis C virus, IQR: interquartile range, MASH: metabolic dysfunction‐associated steatohepatitis, MELD: model for end‐stage liver disease, P‐CAB: potassium‐competitive acid blocker, PPI: proton pump inhibitor, RFX: rifaximin, SAAG: serum–ascites albumin gradient.

The median Child‐Pugh score was 10 (IQR, 9–11); 38 patients (26.8%) were classified as Child‐Pugh class B and 104 patients (73.2%) as class C. The median serum–ascites albumin gradient (SAAG) was 1.9 (IQR, 1.6–2.3) g/dL. Hepatocellular carcinoma (HCC) was present in 41 patients (28.9%). Esophagogastric varices of grade F2 or higher were observed in 84 patients (59.2%).

Regarding the use of acid‐suppressive therapies, 10 patients (7.0%) were receiving H2RA, 100 patients (70.4%) were receiving PPI, and 32 patients (22.5%) were receiving P‐CAB. In terms of the oral environment, 52 patients (36.9%) were wearing dentures, and 5 patients (3.5%) exhibited clinically notable oral contamination based on the OHAT.

Among the total cohort, 47 patients (33.1%) were in the RFX‐treated group, while the remaining 95 patients (66.9%) were in the non‐RFX group. Compared with the non‐RFX group, patients in the RFX‐treated group had a significantly higher median Child–Pugh score (11 vs. 10, *p* = 0.0140), a higher proportion of Child–Pugh class C (87.2% vs. 66.3%, *p* = 0.0087), and a greater proportion with F2 of higher esophagogastric varices (76.7% vs. 50.5%, *p* = 0.0036). The median SAAG was also slightly higher in the RFX‐treated group than in the non‐RFX group (2.0 g/dL vs. 1.9 g/dL, *p* = 0.0755), although the difference did not reach statistical significance. In contrast, there were no significant differences between the two groups in the use of acid‐suppressive medications, frequency of denture use, or presence of oral contamination. A comprehensive summary of the demographic and clinical characteristics is provided in Table 1.

### 3.2 Ascitic Fluid Culture Results at the Onset of SBP

At the initial diagnosis of SBP, ascitic fluid cultures were positive in 71 patients (50.0%). The most frequently isolated organism was *Escherichia coli* (*n* = 19, 26.8%), followed by *Streptococcus salivarius* and *Streptococcus mitis/oralis* (*n* = 5, 7.0%), and *Klebsiella pneumoniae* (*n* = 4, 5.6%) (Table S2). Overall, enteric bacteria accounted for 40 isolates (56.3%), oral bacteria for 26 isolates (36.6%), and skin commensals for 5 isolates (7.0%).

**FIGURE 1:**
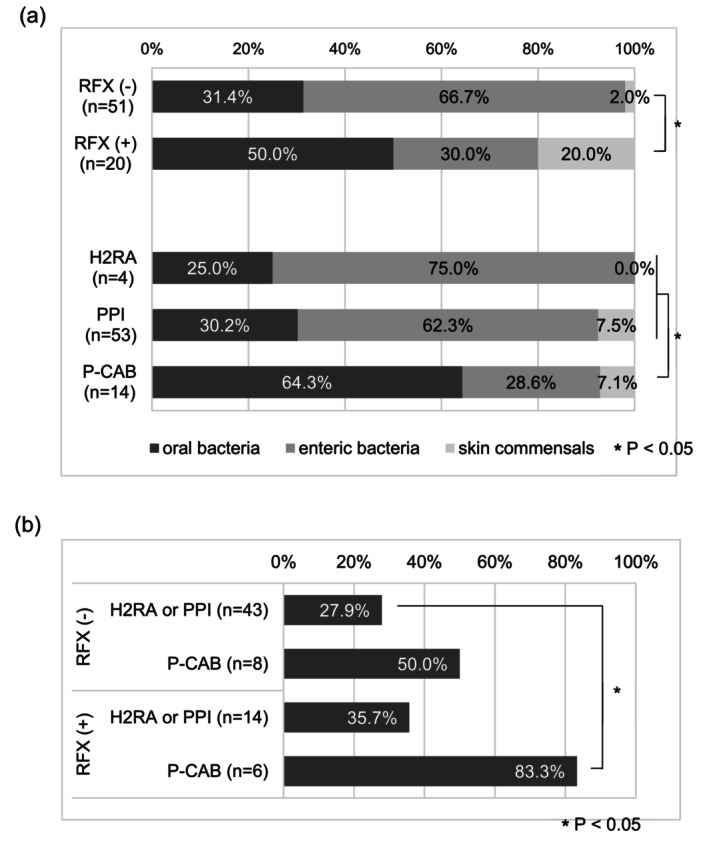
Bacterial species isolated from ascitic fluid at the onset of the first spontaneous bacterial peritonitis episode. (a) Distribution of isolated bacteria according to the presence or absence of rifaximin administration and the type of acid‐suppressive therapy. (b) Frequency of oral bacteria detection based on combinations of rifaximin administration and acid‐suppressive agents. *p < 0.05. Black bars indicate oral bacteria, gray bars indicate enteric bacteria, and light gray bars indicate skin commensals. H2RA: Histamine H2‐receptor antagonist; P‐CAB: Potassium‐competitive acid blocker; PPI: Proton pump inhibitor; RFX: Rifaximin.

Among the non‐RFX group (*n* = 95), ascitic fluid cultures yielded positive results in 51 patients (53.7%): 34 isolates (66.7%) were of enteric bacteria, 16 (31.4%) were oral bacteria, and 1 (1.9%) was skin commensal. In contrast, among the RFX‐treated group (*n* = 47), 20 patients (42.6%) had positive ascitic fluid cultures: 6 isolates (30.0%) were of enteric bacteria, 10 (50.0%) were oral bacteria, and 4 (20.0%) were skin commensals. Although the overall culture positivity rate did not differ significantly between the RFX‐treated group and non‐RFX groups, a significantly lower proportion of enteric bacteria and a higher proportion of oral bacteria were observed in the RFX‐treated group compared to the non‐RFX group (*p* = 0.0033, chi‐squared test) (Figure 1a).

When stratified according to the type of acid‐suppressive therapy, culture positivity was observed in 4 of 10 patients (40.0%) receiving H2RA, in 52 of 100 patients (53.0%) receiving PPI, and in 14 of 32 patients (43.8%) receiving P‐CAB, with no significant differences among the groups. However, among culture‐positive patients, the frequency of oral bacteria was 25.0% (1/4) in the H2RA group, 30.2% (16/53) in the PPI group, and 64.3% (9/14) in the P‐CAB group. Notably, the detection rate of oral bacteria was significantly higher in the P‐CAB group than in the H2RA and PPI groups (*p* = 0.0490, Fisher's exact test). Furthermore, among patients who did not receive RFX but were treated with either H2RA or PPI, the detection rate of oral bacteria was significantly lower than that among patients who received both RFX and P‐CAB (27.9% vs. 83.3%, *p* = 0.0212, Fisher's exact test) (Figure 1b).

### 3.3 Outcome After the First Episode of SBP

**FIGURE 2:**
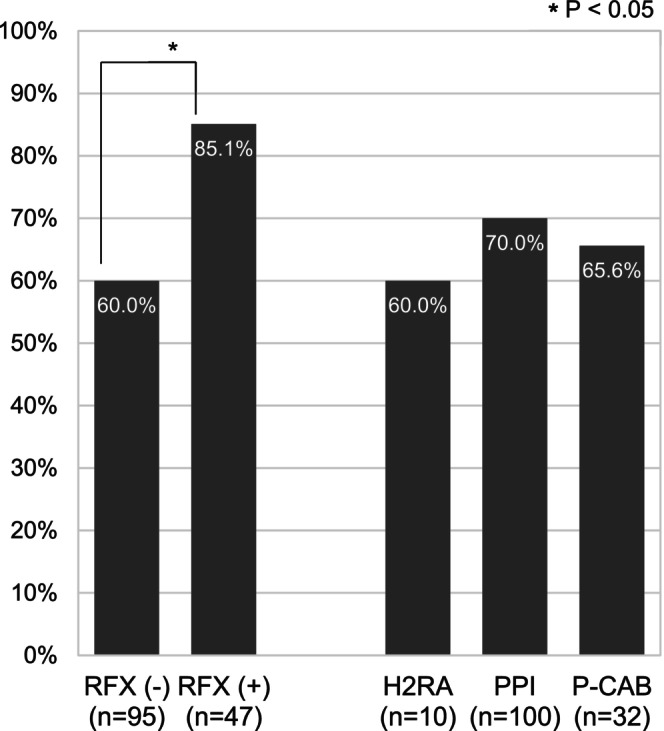
90‐day survival rate after the onset of the first spontaneous bacterial peritonitis episode according to rifaximin administration and the type of acid‐suppressive therapy. *p < 0.05. H2RA: Histamine H2‐receptor antagonist; P‐CAB: Potassium‐competitive acid blocker; PPI: Proton pump inhibitor; RFX: Rifaximin.

Among the 142 patients diagnosed with SBP, 97 patients (68.3%) were alive at 90 days following multidisciplinary treatment. The 90‐day survival rate was higher in patients receiving RFX at SBP onset than in those not receiving RFX (85.1% vs. 60.0%, *p* = 0.0023, Fisher's exact test). In contrast, no significant difference in the 90‐day survival rate was noted among patients according to the type of acid‐suppressive therapy administered: 60.0% (6/10) in the H2RA group, 70.0% (70/100) in the PPI group, and 65.6% (21/32) in the P‐CAB group (*p* = 0.558, Pearson's chi‐squared test) (Figure 2).

Univariate logistic regression analysis identified six variables associated with 90‐day survival (Table 2). RFX administration (odds ratio [OR] 3.81; 95% confidence interval [CI], 1.62–10.08; *p* = 0.0016) and the presence of esophagogastric varices classified as F2 or greater (OR 2.42; 95% CI, 1.18–5.04; *p* = 0.0156) were identified as positive predictors of survival. Conversely, a positive ascitic fluid culture (OR 0.20; 95% CI, 0.08–0.43; *p* < 0.0001), denture use (OR 0.41; 95% CI, 0.20–0.86; *p* = 0.0174), and higher MELD score (per +1 point, OR 0.88; 95% CI, 0.83–0.93; *p* < 0.0001) were identified as negative predictors. A multivariate logistic regression model incorporating these variables revealed four independent predictors of 90‐day survival (Table 3). RFX administration remained independently associated with 90‐day survival (OR 3.62; 95% CI, 1.30–11.28; *p* = 0.0131), while a positive ascitic fluid culture (OR 0.18; 95% CI, 0.06–0.46; *p* = 0.0002), denture use (OR 0.22; 95% CI, 0.07–0.59; *p* = 0.0021), and a higher MELD score (per +1 point, OR 0.84; 95% CI, 0.77–0.90; *p* < 0.0001) were independently associated with lower 90‐day survival (Table 2).

**TABLE 2:** Predictors of 90‐day survival following the first episode of spontaneous bacterial peritonitis.

Variable	Univariate analysis		Multivariate analysis	
	OR (95% CI)	*p*	OR (95% CI)	*p*
Age (per +1 year)	1.00 (0.96–1.02)	0.8123		
Male	1.64 (0.76–3.45)	0.1910		
ALD (vs. others)	0.96 (0.47–2.01)	0.9255		
Child‐Pugh class C (vs. class B)	0.48 (0.19–1.12)	0.0913	—	—
MELD score (per +1 point)	0.88 (0.83–0.93)	< 0.0001	0.84 (0.77–0.90)	< 0.0001
SAAG (per +1 g/dL)	1.91 (0.90–4.22)	0.5233		
RFX use	3.81 (1.62–10.08)	0.0016	3.62 (1.30–11.28)	0.0131
Ascitic fluid culture positive	0.20 (0.08–0.43)	< 0.0001	0.18 (0.06–0.46)	0.0002
P‐CAB use (vs. H2RA/PPI)	0.85 (0.38–2.02)	0.7121		
Denture use	0.41 (0.20–0.86)	0.0174	0.22 (0.07–0.59)	0.0021
Oral contamination present	0.69 (0.11–5.35)	0.6900		
EGV ≧ F2	2.42 (1.18–5.04)	0.0156	—	—
HCC present	0.99 (0.46–2.22)	0.9978		

*Note:* ―: not included in multivariate analysis.

Abbreviations: ALD: alcohol‐associated or ‐related liver disease, F2: form 2 or greater (esophagogastric varices grading), H2RA: histamine H2‐receptor antagonist, HCC: hepatocellular carcinoma, OR: odds ratio, P‐CAB: potassium‐competitive acid blocker, PPI: proton pump inhibitor, RFX: rifaximin, SAAG: serum–ascites albumin gradient, SBP: spontaneous bacterial peritonitis.

**TABLE 3:** Fine–gray subdistribution hazard model for factors associated with recurrence of spontaneous bacterial peritonitis.

Variable	Univariate analysis		Multivariate analysis	
	OR (95% CI)	*p*	OR (95% CI)	*p*
Age (per +1 year)	0.98 (0.96–1.01)	0.1000		
Male	1.88 (0.98–3.62)	0.0580	1.43 (0.70–2.91)	0.3200
ALD (vs. others)	1.76 (0.99–3.11)	0.0530	1.33 (0.73–2.43)	0.3600
Child‐Pugh class C (vs. class B)	0.75 (0.40–1.40)	0.3616		
MELD score (per +1 point)	1.04 (0.99–1.08)	0.1000		
RFX use	0.18 (0.09–0.36)	< 0.0001	0.19 (0.09–0.40)	< 0.0001
P‐CAB use (vs. H2RA/PPI)	0.74 (0.35–1.56)	0.4300		
Denture use	0.70 (0.35–1.39)	0.3100		
Oral contamination present	1.38 (0.40–4.65)	0.6100		
EGV ≧ F2	0.95 (0.53–1.73)	0.8800		
HCC present	0.71 (0.38–1.32)	0.2800		

Abbreviations: ALD: alcohol‐associated or ‐related liver disease, F2: form 2 or greater (esophagogastric varices grading), H2RA: histamine H2‐receptor antagonist, HCC: hepatocellular carcinoma, OR: odds ratio, P‐CAB: potassium‐competitive acid blocker, PPI: proton pump inhibitor, RFX: rifaximin, SAAG: serum–ascites albumin gradient, SBP: spontaneous bacterial peritonitis.

### 3.4 Cumulative SBP Recurrence Rate After Initial Onset

The cumulative recurrence rate of SBP was evaluated in 97 patients who survived the initial episode of SBP. At the time of the first SBP episode, 40 patients were already receiving RFX for hepatic encephalopathy, whereas 57 were not. During a median follow‐up period of 7.7 months, SBP recurred in 47 patients, while 37 patients died during follow‐up (death was treated as a competing event for recurrence). During follow‐up, 8 of 57 patients in the non‐RFX group initiated RFX due to incident hepatic encephalopathy and were censored at RFX initiation in the primary recurrence analysis. Based on Gray's test, with death considered as a competing risk, the cumulative recurrence rates of SBP were 28.2% at 6 months and 41.6% at 12 months.

Potential predictors of SBP recurrence were analyzed using the Fine–Gray sub‐distribution hazard model. In the univariate analysis, RFX administration was significantly associated with a lower cumulative incidence of SBP recurrence (hazard ratio [HR], 0.18; 95% confidence interval [CI], 0.09–0.36; *p* < 0.0001). Male sex (HR, 1.88; 95% CI, 0.98–3.62; *p* = 0.058) and the presence of ALD (HR, 1.76; 95% CI, 0.99–3.11; *p* = 0.053) were marginally associated with increased risk. In the multivariate model including these three variables, RFX administration remained the only independent predictor associated with a significantly lower risk of recurrence (HR, 0.19; 95% CI, 0.09–0.40; *p* < 0.0001) (Table 3). In a sensitivity analysis excluding the 8 crossover patients who initiated RFX during follow‐up, the association between RFX use and lower SBP recurrence remained robust (HR, 0.16; 95% CI, 0.07–0.37; *p* < 0.0001).

**FIGURE 3:**
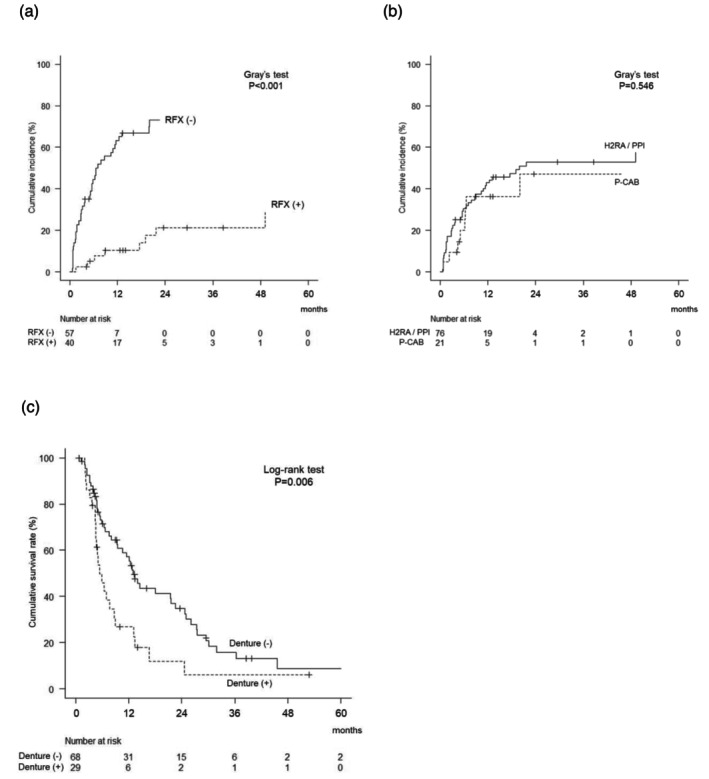
Cumulative recurrence rate and overall survival following the first episode of spontaneous bacterial peritonitis. (a) Cumulative SBP recurrence rates in rifaximin‐treated versus nonrifaximin groups. Patients in the non‐RFX group who initiated RFX during follow‐up (*n* = 8) were censored at the time of RFX initiation. (b) Stratified analysis of SBP recurrence rates by type of acid‐suppressive therapy (H2RA, PPI, P‐CAB). (c) Kaplan–Meier survival curves according to denture use. H2RA: Histamine H2‐receptor antagonist; P‐CAB: Potassium‐competitive acid blocker; PPI: Proton pump inhibitor; RFX: Rifaximin.

Consistently, the cumulative recurrence rate of SBP was significantly lower in the RFX‐treated group (*n* = 40) than in the non‐RFX group (*n* = 57). At 6 and 12 months, recurrence rates in the RFX group were 5.1% and 10.4%, respectively, compared to 44.5% and 63.2% in the non‐RFX group (*p* < 0.001, Gray's test) (Figure 3a). In contrast, the type of acid suppressant used was not associated with SBP recurrence (*p* = 0.546, Gray's test) (Figure 3b).

### 3.5 Cirrhosis‐Related Events and Prognosis Following the Onset of SBP

Among the 97 patients who survived the initial episode of SBP, the incidence of cirrhosis‐related complications and the overall prognosis were investigated (Table S3). During the follow‐up period, overt hepatic encephalopathy developed in 59 patients (60.8%), esophageal variceal rupture occurred in 7 patients (7.2%), and HRS–AKI was observed in 37 patients (38.1%). In total, 37 patients (38.1%) died during the observation period (Table S4).

Using a Fine–Gray regression model with death considered as a competing risk, multivariate analysis revealed that the cumulative incidence of overt hepatic encephalopathy was 34.5% at 6 months and 48.3% at 12 months, and was significantly associated with the presence of oral contamination (hazard ratio [HR] 2.56; 95% confidence interval [CI], 1.32–7.32; *p* = 0.037). The cumulative incidence of esophageal variceal rupture was 11.6% at 6 months and 17.6% at 12 months, and was significantly associated with oral contamination (HR 6.72; 95% CI, 2.13–21.15; *p* = 0.001) and the presence of high‐grade varices (F2 or greater) (HR 4.26; 95% CI, 1.24–14.64; *p* = 0.020). Furthermore, the cumulative incidence of HRS–AKI was 20.2% at 6 months and 31.4% at 12 months, and was significantly associated with oral furosemide administration at doses of 20 mg or more (HR 2.70; 95% CI, 1.31–5.58; *p* = 0.0071) (Table S2). No significant associations were observed between these complications and the use of either rifaximin or acid‐suppressive agents.

The cumulative survival rates were 63.5% at 6 months and 47.6% at 12 months. According to a Cox proportional hazards model, mortality was significantly associated with the presence of hepatocellular carcinoma (HCC) (HR 1.65; 95% CI, 1.01–2.69; *p* = 0.0459), higher MELD scores (per +1 point: HR 1.04; 95% CI, 1.00–1.07; *p* = 0.0329), and denture use (HR 2.00; 95% CI, 1.21–3.31; *p* = 0.0072). In the multivariate analysis, higher MELD score (per +1 point: HR 1.04; 95% CI, 1.01–1.07; *p* = 0.0266) and denture use (HR 2.04; 95% CI, 1.23–3.38; *p* = 0.0057) remained independent predictors of mortality (Table 4). The cumulative survival rates of patients with dentures (*n* = 29) were 46.0% at 6 months and 26.8% at 12 months, while those without dentures (*n* = 68) were 71.4% and 57.1%, respectively. Survival differed significantly according to denture use (*p* = 0.0061, Log‐rank test) (Figure 3c).

**TABLE 4:** Cox proportional hazards analysis of factors associated with mortality following the first episode of spontaneous bacterial peritonitis.

Variable	Univariate analysis		Multivariate analysis	
	HR (95% CI)	*p*	HR (95% CI)	*p*
Age (+1 year)	1.02 (0.99–1.04)	0.09045		
Male sex	1.03 (0.62–1.70)	0.9114		
Etiology: alcohol	0.81 (0.50–1.31)	0.3843		
Rifaximin use	0.76 (0.47–1.22)	0.2503		
P‐CAB use (vs. H2RA/PPI)	0.77 (0.38–1.31)	0.2666		
HCC present	1.65 (1.01–2.69)	0.0459	—	—
Child‐Pugh class C (vs class B)	0.92 (0.55–1.54)	0.7489		
MELD score (+1 point)	1.04 (1.00–1.07)	0.0329	1.04 (1.00–1.07)	0.0266
Denture use	2.00 (1.21–3.31)	0.0072	2.04 (1.23–3.38)	0.0057
Oral contamination present	1.90 (0.59–6.10)	0.2800		
Esophagogastric varices ≧ F2	1.32 (0.79–2.18)	0.2873		

*Note:* —: not included in the multivariate analysis.

Abbreviations: ALD: alcohol‐associated or ‐related liver disease, F2: form 2 or greater (esophagogastric varices grading), H2RA: histamine H2‐receptor antagonist, HCC: hepatocellular carcinoma, HR: hazard ratio, P‐CAB: potassium‐competitive acid blocker, PPI: proton pump inhibitor, RFX: rifaximin, SBP: spontaneous bacterial peritonitis.

## 4 Discussion

In the present study, RFX administration in cirrhotic patients with refractory ascites who developed SBP was associated with multiple favorable outcomes: reduced isolation of enteric bacteria at SBP onset, improved 90‐day survival, and a significantly lower risk of recurrence among survivors. These benefits were observed despite the RFX‐treated group having more severe baseline liver disease, including higher Child–Pugh scores and more advanced variceal grades, indicating that the associations are unlikely to be explained solely by baseline disease severity. An additional novel finding was the identification of oral contamination at admission as a prognostic factor for subsequent cirrhosis‐related complications, particularly overt hepatic encephalopathy and variceal bleeding, thereby providing associative signals that may support the concept of the oral–gut–hepatic axis. Furthermore, denture use was associated with increased mortality, suggesting that oral health status, within the context of the oral–gut–hepatic axis and often underappreciated in hepatology practice, may play a meaningful role in patient outcomes.

The prophylactic efficacy of RFX against SBP has been reported in several international studies [8, 15, 16, 17], which have generally concluded that RFX reduces the risk of SBP occurrence and recurrence through modulation of the gut microbiota, suppression of bacterial overgrowth, and inhibition of bacterial translocation. Our results are consistent with these findings and extend them to a Japanese population, where routine use of prophylactic antibiotics for SBP remains limited due to health insurance restrictions. The magnitude of benefit in our cohort, both in terms of short‐term survival and recurrence prevention, reinforces the clinical relevance of RFX in high‐risk cirrhotic patients with refractory ascites.

Notably, microbiological analyses in this study revealed that patients in the RFX‐treated group had a lower proportion of enteric bacteria in ascitic cultures compared with those in the non‐RFX group, with a relative enrichment of oral bacteria. Importantly, this finding should be interpreted as a relative shift in the distribution of detected organisms rather than an absolute increase in oral bacterial burden. Rifaximin likely suppresses enteric bacteria, thereby increasing the relative proportion of oral bacteria among culture‐positive ascitic fluid samples. A particularly novel finding of the present study is the demonstration that the microbial profile of SBP in patients with cirrhosis reflects not only intestinal but also oral bacteria, thereby providing clinical evidence to support the concept of an “oral–gut–hepatic axis” [10, 11]. Although previous investigations have primarily focused on gut‐derived bacterial translocation as the dominant pathogenic mechanism of SBP, our results suggest that suppression of enteric bacteria by rifaximin may unmask the contributory role of oral bacteria. This microbial shift, together with the observed associations between oral contamination, denture use, and adverse clinical outcomes, highlights the oral cavity as a previously underrecognized source of peritoneal infection in patients with cirrhosis. These findings extend the current paradigm of SBP pathogenesis beyond the gut and suggest that measures to improve oral hygiene should be considered in conjunction with intestinal decontamination strategies. Thus, the present study provides the first real‐world evidence that the oral–gut–hepatic axis constitutes not only a theoretical framework but also a clinically significant pathway influencing the prognosis of cirrhotic patients with SBP.

Our findings regarding oral contamination and its impact on prognosis align with previous studies showing that periodontal care reduces the risk of hepatic encephalopathy and infectious complications in cirrhosis [12, 18, 19]. Possible mechanisms include bacterial dissemination from periodontal lesions into the systemic circulation, increased inflammatory burden, and facilitation of bacterial translocation through swallowing of oral microbiota. Denture use may contribute by providing a surface for biofilm formation, harboring pathogenic bacteria, and potentially serving as a reservoir for infection. Moreover, denture wearers may represent a frailer subset of patients with poorer nutritional and functional status, which could partly explain the association with mortality.

RFX is a nonabsorbable, broad‐spectrum antibiotic with activity against both gram‐positive and gram‐negative aerobic and anaerobic bacteria. By reducing intestinal bacterial overgrowth and suppressing ammonia‐producing organisms, it not only lowers systemic ammonia levels but also reduces the risk of peritoneal contamination by enteric bacteria. In contrast, oral contamination, particularly in the setting of impaired gastric acid barriers due to P‐CAB or PPI use, may enable oral bacteria to survive gastrointestinal transit, increasing the likelihood of peritoneal seeding by oral bacteria. This mechanistic explanation may account for our observation that in RFX‐treated patients, the relative proportion of oral bacteria in ascitic fluid increased despite suppression of enteric bacteria. In this context, recent next‐generation sequencing studies have shown that ascitic fluid, including culture‐negative samples, often contains detectable bacterial DNA and a diverse microbial community that may not be recovered by conventional culture methods. These findings underscore the limitations of culture‐based classification and highlight the value of culture‐independent microbiome analyses for future investigations.

This study has several limitations. First, it was a single‐center, retrospective observational study, and patient selection or treatment policies may have been influenced by facility‐specific factors; validation in multicenter studies is warranted. Second, microbiological assessment relied on conventional culture methods without the use of culture‐independent techniques such as next‐generation sequencing, potentially underestimating the diversity of SBP‐related microbiota. Third, oral contamination was evaluated retrospectively based on medical and nursing records rather than by a standardized prospective dental examination. Although oral status is routinely assessed by nursing staff at admission using an OHAT‐based framework, oral contamination in this study was defined by the presence of documentation indicating that oral care was required, and formal OHAT scores were not calculated. Consequently, this chart‐based reconstruction may have underestimated the prevalence and severity of oral health impairment, and findings related to oral contamination should be interpreted as hypothesis‐generating. Finally, RFX prophylaxis is not covered by health insurance in Japan, and prescribing practices depend on physician discretion and patient circumstances, which may have introduced selection bias and unmeasured confounding, including confounding by indication. Moreover, because some patients initiated RFX during follow‐up due to changes in clinical status (e.g., incident hepatic encephalopathy), censoring at the time of RFX initiation in the recurrence analysis could be informative; therefore, residual bias cannot be fully excluded. Given the exploratory nature of multiple group‐wise comparisons without multiplicity adjustment, findings with borderline *p* values should be interpreted cautiously. In addition, although the presence of hepatocellular carcinoma was included in the survival analysis, detailed tumor staging (e.g., BCLC stage) at the time of SBP onset could not be consistently assessed in this retrospective cohort and was not incorporated, which may have resulted in residual confounding. Future multicenter, prospective studies with microbiome analyses are needed to confirm these findings and to establish effective preventive strategies integrating intestinal and oral interventions for SBP in cirrhosis.

In conclusion, RFX administration was associated with favorable microbiological and clinical outcomes in Japanese cirrhotic patients with refractory ascites who developed SBP. Oral contamination and denture use were identified as potential adverse prognostic factors, providing clinical evidence for the oral–gut–hepatic axis and supporting the integration of oral hygiene assessment and management into SBP prevention strategies. These results suggest that combining intestinal‐targeted therapy with oral care, within the framework of the oral–gut–hepatic axis, may represent a comprehensive approach to reducing SBP recurrence and improving prognosis.

## Funding

The authors have nothing to report.

## Conflicts of Interest

Satoshi MOCHIDA has received speaking fees or honoraria from Abbvie GK, Gilead Sciences Inc., ASKA Pharmaceutical Co. Ltd., AstraZeneca K.K., Ohtsuka Pharmaceutical Co. Ltd., Torey Medical Co. Ltd., Eisai Co. Ltd., Chugai Pharmaceutical Co. Ltd., has received research grants from Abbvie GK, Eisai Co. Ltd. Sumitomo Pharma Co. Ltd. intellim Corporation.

## Supporting information


**Table S1:** Classification of bacterial species isolated from ascitic fluid.
**Table S2:** Distribution of bacterial isolates from ascitic fluid cultures at the onset of spontaneous bacterial peritonitis (*n* = 71).
**Table S3:** Additional baseline characteristics of patients included in the prognostic analyses at the onset of spontaneous bacterial peritonitis.
**Table S4:** Predictors of cirrhosis‐related complications among survivors after the first episode of spontaneous bacterial peritonitis.

## Data Availability

The data that support the findings of this study are available from the corresponding author upon reasonable request.
