# Supplementary material for: Rifaximin Use Is Associated With Prognosis and Recurrence Patterns in Spontaneous Bacterial Peritonitis: Exploratory Insights Into the Oral–Gut–Hepatic Axis
Source: JGH Open. 2026 Apr 2;10(4):e70391. doi: 10.1002/jgh3.70391 (PMC13045416; doi:10.1002/jgh3.70391)
Supplement: Supplementary file 1 — Table S1: Classification of bacterial species isolated from ascitic fluid. Table S2: Distribution of bacterial isolates from ascitic fluid cultures at the onset of spontaneous bacterial peritonitis (n = 71). Table S3: Additional baseline characteristics of patients included in the prognostic analyses at the onset of spontaneous bacterial peritonitis. Table S4: Predictors of cirrhosis‐related complications among survivors after the first episode of spontaneous bacterial peritonitis. [file JGH3-10-e70391-s001.docx]

***Supplementary Table 1. Classification of Bacterial Species Isolated from Ascitic Fluid***

| Bacterial species | Category | Rationale (primary ecological niche) |
| --- | --- | --- |
| Escherichia coli | Enteric | Predominant intestinal commensal (Enterobacterales) |
| Klebsiella pneumoniae | Enteric | Gut-derived Enterobacterales |
| Klebsiella oxytoca | Enteric | Gut-derived Enterobacterales |
| Klebsiella variicola/pneumoniae | Enteric | Gut-derived Enterobacterales |
| Enterobacter cloacae | Enteric | Gut-derived Enterobacterales |
| Citrobacter freundii complex | Enteric | Intestinal commensal |
| Serratia marcescens | Enteric | Intestinal commensal |
| Proteus mirabilis | Enteric | Intestinal commensal |
| Enterococcus faecalis | Enteric | Intestinal commensal |
| Pseudomonas aeruginosa | Enteric | Gastrointestinal colonizer in cirrhosis |
| Achromobacter xylosoxidans | Enteric | Opportunistic gastrointestinal colonizer |
| Streptococcus salivarius | Oral | Oral commensal (viridans group) |
| Streptococcus mitis/oralis | Oral | Viridans group streptococci, oral cavity |
| Streptococcus mitis group | Oral | Viridans group streptococci |
| Streptococcus parasanguinis | Oral | Oral commensal |
| Streptococcus gordonii | Oral | Oral commensal |
| Streptococcus vestibularis | Oral | Oral commensal |
| Streptococcus cristatus | Oral | Oral commensal |
| Streptococcus oralis | Oral | Oral commensal |
| Streptococcus anginosus group | Oral | Oral–oropharyngeal commensal |
| Streptococcus pneumoniae | Oral | Oropharyngeal commensal |
| Streptococcus agalactiae (GBS) | Oral* | Oropharyngeal colonizer in adults |
| Streptococcus gallolyticus ssp. pasteurianus | Oral* | Oropharyngeal / GI commensal (classified as oral in this study) |
| Staphylococcus epidermidis | Skin commensal | Cutaneous flora |
| Staphylococcus aureus | Skin commensal | Cutaneous flora |
| Staphylococcus caprae (MRCNS) | Skin commensal | Cutaneous flora |
| Candida tropicalis | Fungal isolate | Non-bacterial organism (excluded from bacterial classification analyses) |

* Classified as oral flora in this study based on predominant oropharyngeal colonization in adults, acknowledging possible gastrointestinal overlap.

***Supplementary Table 2. Distribution of Bacterial Isolates from Ascitic Fluid Cultures*** ***at the Onset of Spontaneous Bacterial Peritonitis (n = 71)***

| Bacterial Species | n (%) |
| --- | --- |
| *Escherichia coli* | 19 (26.8%) |
| *Streptococcus salivarius* | 5 (7.0%) |
| *Streptococcus mitis/oralis* | 5 (7.0%) |
| *Klebsiella pneumoniae* | 4 (5.6%) |
| *Enterococcuc faecalis* | 3 (4.2%) |
| *Streptococcus parasanguinis* | 3 (4.2%) |
| *Citrobacter freundii complex* | 3 (4.2%) |
| *Enterobacter cloacae* | 2 (2.8%) |
| *Klebsiella oxytoca* | 2 (2.8%) |
| *Serratia marcescens* | 2 (2.8%) |
| *Staphylococcus epidermidis* | 2 (2.8%) |
| *Staphylococcus aureus* | 2 (2.8%) |
| *Streptococcus agalactiae（GBS)* | 2 (2.8%) |
| *Streptococcus gordonii* | 2 (2.8%) |
| *Streptococcus mitis group* | 2 (2.8%) |
| *Streptococcus pneumoniae* | 2 (2.8%) |
| *Achromobacter xylosoxidams subsp xylosoxidans* | 1 (1.4%) |
| *Candida tropicalis* | 1 (1.4%) |
| *Klebsiella variicola/pneumoniae* | 1 (1.4%) |
| *Proteus mirabilis* | 1 (1.4%) |
| *Pseudomonas aeruginosa* | 1 (1.4%) |
| *Staphylococcus caprae MRCNS* | 1 (1.4%) |
| *Streptococcus cristatus* | 1 (1.4%) |
| *Streptococcus oralis* | 1 (1.4%) |
| *Streptococcus anginosus group* | 1 (1.4%) |
| *Streptococcus gallolyticus ssp pasteurianus* | 1 (1.4%) |
| *Streptococcus vestibularis* | 1 (1.4%) |

***Supplementary Table 3. Additional Baseline Characteristics of Patients Included in the Prognostic Analyses at the Onset of Spontaneous Bacterial Peritonitis***

| Factors | n (%), median (IQR) |
| --- | --- |
| Renal function | |
| Serum creatinine, mg/dL | 1.09 (0.78 – 1.80) |
| eGFR, mL/min/1.73 m^2^ | 49.3 (30.1 – 70.4) |
| Diuretic use | |
| Furosemide dose, mg/day  0  10  ≥20 | 61 (62.9%)  24 (24.7%)  12 (12.4%) |
| Spironolactone dose, mg/day  0  25  50  75  ≥100 | 8 (8.2%)  31 (32.0%)  48 (49.5%)  8 (8.2%)  2 (2.1%) |
| Tolvaptan dose, mg/day  0  3.25  7.5 | 12 (12.4%)  1 (1.3%)  84 (86.6%) |
| Hepatocellular carcinoma | |
| Absent  Present | 69 (71.1%)  28 (28.9%) |

eGFR, estimated glomerular filtration rate

Only patients included in the prognostic analyses were analyzed in this table.

***Supplementary Table 4. Predictors of Cirrhosis-related Complications among Survivors after The First Episode of Spontaneous Bacterial Peritonitis***

| Event | n (%) | Cumulative incidence | | Risk factor | Multivariate analysis | |
| --- | --- | --- | --- | --- | --- | --- |
|  |  | at 6 months (%) | at 12 months (%) |  | HR (95% CI) | P-value |
| Overt hepatic encephalopathy | 59 (60.8) | 34.5 | 48.3 | Oral contamination | 2.56 (1.32 – 7.32) | 0.0370 |
| Esophageal variceal rupture | 7 (7.2) | 11.6 | 17.6 | Oral contamination | 6.72 (2.13 – 21.15) | 0.0010 |
|  |  |  |  | High-grade varices | 4.26 (1.24 – 14.64) | 0.0200 |
| HRS-AKI | 37 (38.1) | 36.5 | 52.4 | Furosemide ≥20 mg/day | 2.70 (1.31 – 5.58) | 0.0071 |

AKI: acute kidney injury, HR: hazard ratio, HRS: hepatorenal syndrome
